# Intestinal Parasites of Owned Dogs and Cats from Metropolitan and Micropolitan Areas: Prevalence, Zoonotic Risks, and Pet Owner Awareness in Northern Italy

**DOI:** 10.1155/2014/696508

**Published:** 2014-04-28

**Authors:** Sergio Aurelio Zanzani, Alessia Libera Gazzonis, Paola Scarpa, Federica Berrilli, Maria Teresa Manfredi

**Affiliations:** ^1^Department of Veterinary Science and Public Health, Università degli Studi di Milano, 20133 Milan, Italy; ^2^Department of Public Health, Università di Tor Vergata, 00133 Rome, Italy

## Abstract

Intestinal parasites of dogs and cats are cosmopolitan pathogens with zoonotic potential for humans. Our investigation considered their diffusion in dogs and cats from northern Italy areas, specifically the metropolitan area of Milan and two micropolitan areas of neighboring provinces. It included the study of the level of awareness in pet owners of the zoonotic potential from these parasites. A total of 409 fresh fecal samples were collected from household dogs and cats for copromicroscopic analysis and detection of *Giardia duodenalis* coproantigens. The assemblages of *Giardia* were also identified. A questionnaire about intestinal parasites biology and zoonotic potential was submitted to 185 pet owners. The overall prevalence of intestinal parasites resulted higher in cats (47.37%−60.42%) and dogs (57.41%−43.02%) from micropolitan areas than that from the metropolis of Milan (dogs: *P* = 28.16%; cats: *P* = 32.58 %). The zoonotic parasites infecting pets under investigation were *T. canis* and *T. cati*, *T. vulpis*, Ancylostomatidae, and *G. duodenalis* assemblage A. Only 49.19% of pet owners showed to be aware of the risks for human health from canine and feline intestinal parasites. Parasitological results in pets and awareness determination in their owners clearly highlight how the role of veterinarians is important in indicating correct and widespread behaviors to reduce risks of infection for pets and humans in urban areas.

## 1. Introduction

Intestinal parasites of dogs and cats are diffused worldwide. Though some differences can be noticed between stray and shelter dogs, and even in pets in general, veterinarian concern for these parasites is still living matter due to their zoonotic potential and their significant pathogen effects on carnivore hosts [1]. The overall prevalence of intestinal parasites in pet dogs and cats varies considerably. In fact, recent studies revealed percentages from 12.5% to 34.4% in dogs and from 10.1% to 22.8% in cats. High variability also relates to single species or taxon [2–6].* Giardia duodenalis* appeared to be the most frequent parasite with prevalence values varying from 1.3% to 24.78% (dogs) and from 0% to 20.31% (cats) [5, 7–10]. As regards helminthic infections, hookworms, ascarids, and whipworms were the most frequent intestinal parasites in dogs [2, 3, 5, 6, 8, 10–12]. In cats,* Toxocara cati* was the most common helminth with prevalence values ranging from 1.5% to 10% [7, 8, 10, 13].

Several causes might have affected observed variability in intestinal parasite infections, such as host individual features, management, heartworm prophylactic treatments, and diagnostic techniques [2, 5–7, 9, 11, 14, 15]. Further, canine and feline helminths are susceptible to the effects of environmental condition and to climate change due to their developmental stages and their survival periods in the environment [1, 16, 17].

To date, domestic carnivores still represent an important source of zoonotic helminths, among which the most common* Toxocara* species are far-back well known as regards their impact on human health [1, 18–21]. As to* Giardia, *several surveys showed that carnivore pets host-specific (C, D, F) or zoonotic assemblages (A and B) of* G. duodenalis*, whose prevalence values strongly depend on the diagnostic techniques used; for example, PCR and antigen detection seemed more sensitive than copromicroscopic analysis [10, 22–24].

Lombardy is the region of northwestern Italy with the largest population of companion animals representing about 15% of their overall presence in Italy (data from National Companion Registry http://www.salute.gov.it/anagcaninapublic_new/AdapterHTTP). Nevertheless, only few data are available on the occurrence of intestinal parasites in companion animals in this area. Moreover, they are not updated or just limited to stray cats [25, 26]. Major aim of this survey was to determine the prevalence of intestinal parasites in three groups of dogs and cats from the metropolitan area of Milan and two micropolitan areas at the boundaries of two neighboring provinces where pets can have access or live outdoor more easily than those in Milan. Influence on prevalence of individual pet features (age, gender, size, and presence/absence of clinical signs) and management (household/outdoor, husbandry, and heartworm effective treatment) were also considered. Furthermore, owner awareness mainly about zoonotic potential of intestinal parasites affecting their pets was investigated by a specifically designed questionnaire.

## 2. Materials and Methods

### 2.1. Study Area and Sampling

The survey was carried out in the northwestern Italian region of Lombardy (latitude: 45°40′N; longitude: 9°30′E). Climate is mainly continental except above 1500 m a.s.l. where the typical features of alpine areas are recognized. Consequently, temperatures show high variability with a maximum/minimum annual mean of 35/0°C (even less in the Alpine areas). The mean annual rainfall is 600–700 mm in the southern planes and 2000 mm in the Alpine or Prealpine areas.

The study included dogs and cats from three major provinces of Lombardy: Milan (latitude: 45°30′N, longitude: 9°30′E), Bergamo (latitude: 45°50′N, longitude: 09°48′E), and Brescia (latitude: 45°55′N, longitude: 10°15′E). A total of 202 and 207 fecal samples were collected from owned pets in the metropolitan area of Milan (MT) and in the micropolitan areas of Bergamo (MC 1) and Brescia (MC 2), respectively. In the latter areas, cities with 10000 to 50000 residents were included (MC 1: 23; MC 2: 15). From January 2010 to October 2011, a total of 409 fresh fecal samples were collected by owners or veterinarians from household dogs (*n* = 253) and cats (*n* = 156) that underwent clinical examination in two different veterinary clinics located in the named areas. At clinical examination, data about individual features (age, sex, breed, and presence/absence of clinical signs) and management (indoor/outdoor housing, cohabitation with other dogs and/or cats, and effective prophylaxis against* Dirofilaria immitis* in dogs and in cats older than 12 months) of animals were recorded by clinicians. Further, data (gender, age, education level, and number of family components) about 207 owners were obtained.

### 2.2. Fecal Examination

Feces were stored at +4°C and examined within 48 hours. Macroscopic examination was firstly performed for the detection of proglottids of cestodes. Subsequently, each fecal sample was divided into two aliquots. In order to detect parasite eggs and oocysts one aliquot was subjected to microscopic analysis by centrifugation-flotation technique with sucrose and sodium nitrate solution (specific gravity: 1360). The parasite eggs were differentiated according to their morphologic characteristics. Quantitative measurement of helminth infection (EPG) was not implemented. The second aliquot was used to detect coproantigens of* G. duodenalis *by a commercially available immunochromatographic test (RIDA QUICK* Giardia* cassette, R-Biopharm AG, Germany).

### 2.3. PCR Assay

A group of selected* Giardia*-positive samples were processed by a commercial kit (QIAamp DNA Stool Mini Kit, QIAGEN, Valencia, CA, USA) for DNA extraction. A nested PCR protocol was applied to amplify a fragment of the small subunit ribosomal RNA (SSUrRNA)* Giardia* gene. For external PCR, the forward primer RH11 (5′-CATCCGGTCGATCCTGCC-3′) and the reverse primer RH4 (5′-AGTCGAACCCTGATTCTCCGCCCAGG-3′) designed by Hopkins et al. [27] were used; the internal primers (GIAR-F forward: 3′-GACGCTCTCCCCAAGGAC-5′ and GIAR-R reverse: 5′-CTGCGTCACGCTCG-3′-) designed by Read et al. [28] were used. Amplification products were run on 2% ethidium bromide agarose gels and visualized under ultraviolet light. Bands were excised from agarose gels and purified using a QIAquick Gel Extraction Kit (QIAGEN, Valencia, CA, USA). Amplification products were sent to an external laboratory for sequencing; BLAST analysis of the GeneBank database was performed to identify* G. duodenalis* assemblages from obtained sequences.

### 2.4. Questionnaire Survey

A questionnaire designed to know owner general information on canine and feline intestinal parasites together with their awareness of risks for animal and human health from these parasites was submitted. A total of 185 questionnaires were answered, namely, by 125 dog owners and 60 cat owners.

### 2.5. Statistical Analysis

We defined prevalence after Bush et al. [29]. Prevalence of each parasite within categories of the considered variables was compared using a Chi-Square test and results were retained significant when the null hypothesis had a probability less than *P* < 0.01 or *P* < 0.05. Since prevalence of single taxon was too low for a risk factor analysis, data on infection with helminths and/or* Giardia* were also combined to the purpose. Labelling an animal as positive if tested positive for at least one species of parasite, preliminary univariate logistic regression was performed considering the following independent variables: gender, age (≤12 months old, >12 months old), habitation (metropolitan area and micropolitan areas 1 and 2), management (outdoor or household), breed size (small, medium or large), and cohabitation with other animals. Variables showing a *P* value < 0.20 were included in the multivariate regression model. Backward elimination was used to determine which variables entered the final model, setting at 0.05 the level of significance to be included in the model. The association between infection and gastrointestinal symptoms was analyzed by Chi-Square test. Further, the owner features (gender, age, educational qualification, family components, and presence of young <15 years old) were compared for “infected” or “not infected” pets by Chi-Square test. All statistical analysis was performed using SPSS v.19.0 (IBM Corp., Armonk, NY, USA).

## 3. Results

### 3.1. Parasitological Analysis

The overall prevalence of intestinal parasites resulted higher in dogs and cats from micropolitan areas (dogs: *P* = 57.41% and *P* = 43.02%, resp.; cats: *P* = 47.37% and *P* = 60.42%, resp.) than those from the metropolitan area of Milan (dogs: *P* = 28.16%; cats: *P* = 32.58%). In general, a scarce parasitofauna was detected in most cases of dogs and cats. They were frequently infested by one parasite species (dogs: *P* = 77.94%; cats: *P* = 73.68%) or by two parasite species (dogs: *P* = 22.06%; cats: 26.32%). In both dogs and cats,* G. duodenalis* was the most prevalent species detected. Itsprevalence values accounted as follows: dogs: 20.37% (MC 1) and 25.58% (MC 2), 16.05% (MT); cats: 36.84% (MC 1), 25.00% (MC 2) and 24.7% (MT).* T. canis* resulted to be the most common helminth in dogs from MC 1 (*P* = 22.22%), with a lower prevalence in those from MC 2 (*P* = 9.30%) and MT (*P* = 4.48%)*. T. cati* showed its highest prevalence values in cats from MC 2 (*P* = 22.39%), and lower values in those from MC 1 (5.26%) and MT (*P* = 5.62%) (Table 1).

Considering the univariate logistic regression analysis, in dogs, pet age was the strongest predictor of intestinal parasite infection; the odds of a dog being infected were 0.44 smaller in animals >12 months old (Table 2). Dogs from the metropolitan area of Milan were significantly less susceptible to intestinal parasites than dogs from MC 2 (OR = 1.947) or MC 1 (OR = 3.476) (Table 2). Besides, husbandry management (single or multiple animals in the same house) had impressive effect on the infection: multiple dogs showed higher infection risk than single dogs (OR = 2.059). Gender, breed size, and housing management (household or outdoor) had no impressive effect on the infection. In cats, the predominant predictors of intestinal parasite infection were habitation, age, and housing. Specifically, older cats were less likely infected than younger ones (OR = 0.347), and cats from the metropolitan area of Milan showed less susceptibility to infections than cats from the micropolitan areas (OR = 2.100 and OR = 3.561) (Table 2). The age and husbandry variables for dogs and habitation and age for cats entered in the final multivariable model (Table 3).

Subsequently, data of each taxon was analyzed; dogs <12 months old showed significantly a higher prevalence of infection by* T. canis* (*P* = 20.65%),* Cystoisospora* (8.70%), and by* Giardia duodenalis *(*P* = 27.17%) than older ones (Table 4).* Cystoisospora* was more commonly found in household dogs than in dogs living outdoor. Dogs with multiple husbandries were frequently infected by Ancylostomatidae and* T. vulpis* (Table 4).

In cats,* T. cati* and* T. leonina* infection prevalence resulted significantly higher in young animals than in adult ones.* T. leonina* was more commonly found in cats living outdoor than in household cats (Table 4).

The dogs and cats in this study were presented to two Veterinary Clinics for routine control or vaccination; clinical findings were absent in most cases, except for 20.71% of dogs and 13.43% of cats with gastrointestinal signs, such as diarrhea, vomiting, nausea, or lack of appetite. Out of them, 44.83% of dogs and 44.44% of cats had intestinal parasites (Table 5). A large percentage of the sampled dogs received regular prophylaxis against* D. immitis* (*P* = 70.42%) with selamectin in spot-on formulation (54.17%), ivermectin (39.58%) per os, or moxidectin (6.25%) in injectable formulation. Thus, only a few dogs were infected by helminths. In particular, dogs under selamectin treatment were infected with* T. vulpis* (*n* = 4), Ancylostomatidae (*n* = 2),* T. canis* (*n* = 1), and* Toxascaris leonina* (*n* = 1); dogs under ivermectin treatment were infected with* T. canis* (*n* = 2) and* T. vulpis* (*n* = 1); one dog under moxidectin treatment was infected with* T. canis*.

Only 2 adult cats received proper prophylaxis against heartworms with a spot-on formulation containing selamectin: one of them were infected by* Dipylidium caninum*.

### 3.2. Genotyping of Giardia Duodenalis

Fifty-four* Giardia*-positive samples (37 dogs and 17 cats) were processed for the nested PCR protocol. In dogs, prevalence of* G. duodenalis *assemblages, obtained from 11/37 dogs, showed the occurrence of C and D assemblages, precisely with percentages of 54.5 (C) and 45.45 (D). In cats, A and D assemblages were detected, with percentages of 83.3% (A) and 16.6% (D).

### 3.3. Survey on Health Risk Awareness in Pet Owners

A specifically designed questionnaire on health risk awareness was handed out among owners whose pets were under our investigation. Results from filled-in forms showed that 71.89% of them correctly identified the common transmission route of intestinal parasites, that is, fecal contamination of food or of other ingested materials. While 9.73% of them thought that direct contact between healthy and infected animal triggers infection, 18.38% totally ignored the way of transmission of intestinal parasites. 60.90% of owners identifying in fecal contamination the route of infection for dogs and cats retained that parasite eggs could stay infective for long. About the possibility of transmission of intestinal parasites to puppies/kittens by bitches/queens, only 48.11% of owners answered affirmatively, while 15.67% of them had no answer. When asked about human health risks due to canine and feline intestinal parasites, 49.19% showed awareness of the occurrence, 35.67% answered that no risk is given, and 15.14% declared they had even never considered such probability. Of the ninety owners aware of zoonoses risk, 72.52% of them thought that the most common source of infection is contaminated food, 23% answered that transmission of parasites to humans is caused by direct contact with pets while 4.40% had no idea. Gender, age, and education level of pet owners as well as their family size and possible presence of young members do not seem to affect animal occurrence of intestinal parasite infections (Table 6).

## 4. Discussion

The intestinal parasites in this survey are consistent with the typical parasite spectrum of domestic carnivores worldwide. Among the recovered helminthic species,* T. canis* and* T. cati*, which accountedthe most frequent, are considered of great public health significance in their causing the most widespread and economically important zoonoses [30]. Other parasites diffusing zoonoses of minor importance were found, such as* T. vulpis*, Ancylostomatidae, and* Dypilidium canimum*. Finally, molecular analysis on fecal samples demonstrated the presence of* G. duodenalis* Assemblage A, considered to have zoonotic potential [31]. The overall prevalence of intestinal parasites both in dogs and in cats in northern Italy was higher than expected (*P* = 28.16–57.41% in dogs; *P* = 32.58–60.42% in cats). The currently reported prevalence rates of dog parasites are slightly different considering the different origin of sampled dogs. Particularly, dogs from the large metropolitan area of Milan showed lower prevalence than dogs from the micropolitan territories. Several factors can justify these differences. In fact, most dogs from Milan were rarely taken to large playgrounds, limited in their walks, and regularly treated against heartworm disease. Further, no colonies of stray dogs exist, and proper disposal of dog waste from public soil is coming into common use among urban pet owners. On the other hand, dogs from micropolitan areas are usually at high risk of infection being frequently outdoors in their gardens or in large green areas. In addition, transhumance being still practiced in Lombardy, they might reasonably be infected by sheepdogs guarding transhumant sheep flocks. In fact, they are moved from Alpine pastures to lowlands twice yearly along the main routes (north to south) through Bergamo and Brescia towards the Po Plain areas whose fields may be contaminated by feces of untreated sheepdog, thus passing infection.

In dogs, as regards helminths only, currently reported prevalences significantly differ from what was previously observed in Lombardy. In 1974, in a coprological survey conducted in some micropolitan areas located north and south of Milan, helminths were recorded in 75.79%–85.3% of examined dogs [25]. Further development in diagnosis and treatment may account for the substantial differences found with our present survey together with a more widespread prophylaxis against* D. immitis* in the area of Milan, which might have reasonably contributed to control canine intestinal parasites. In 2007, helminth eggs were recovered in 7% of dog feces collected from public places, including parks, of Milan [32], indicating a lower prevalence than in our latest survey (*P* = 14.1%). This could be due to the kind of fecal samples collected from city soil that mainly included droppings voided by old dogs typically showing lower infection values than young ones. On the other hand, our findings are consistent with results from a recent survey on pets from central Italy sampled in veterinary clinics where helminth infections were present in 24.1% of owned dogs and in 31.9% of owned cats [33]. In the same year, helminth infections were recorded in 50.1% of stray cats from colonies pertaining to the metropolitan area of Milan [26].

Consistent with data obtained in several countries, ascarids, especially* Toxocara *spp., were the most prevalent canine and feline parasites [7, 10, 12–14, 33, 34]. In contrast with other surveys, a low prevalence of Ancylostomatidae infection was recorded in our sampled dogs except those from the micropolitan areas 1 and 2. A low presence of hunting, sporting, or guard dogs in our samples as well as epidemiology and life cycle of Ancylostomatidae nematodes can account for this discrepancy [2, 3, 8, 11, 35].

As regards* G. duodenalis*, it was the most prevalent canine and feline parasite according to other surveys [5, 8–10, 12]. Such findings are not consistent with low prevalence values recorded in the same species in other Italian studies whose analytical methods were different [32, 36–39].

In this survey, consistent with previous studies [31, 40, 41],* G. duodenalis* assemblages C and D were isolated in dogs. They are considered host-adapted genotypes and a species name,* Giardia canis*, was proposed to label them. As regards cats, in our study the host-adapted F genotype was not found; however,* G. duodenalis* infections sustained also by assemblages A, B, C, and D have been previously described in cats [40, 42]. In owned cats, we observed a high prevalence of* G. duodenalis* infection by assemblage A, whose possible zoonotic potential must not be underestimated [31]. Finally,* G. duodenalis* assemblage D was recovered, less frequently though [42, 43].

Risk factors for dogs from metropolitan and micropolitan areas were being younger than 12 months or sharing the same house with other dogs. Compared to dogs from the large metropolitan area of Milan, the odds for dogs from the micropolitan area 1 were 3.476 times higher, and the odds for dogs from MC 2 were 1.947 times higher but with lower significance (*P* = 0.001* versus P* = 0.055). Further, compared to dogs ≤12 months old, the odds of a dog >12 months being parasitized were 0.362 times smaller. Compared to single- household dogs, the odds for multiple- household dogs were 2.059 times higher, which means that cohabitation is one of the most important risk factors associated to endoparasitism. In accordance with Katagiri and Oliveira-Sequeira [2], who also found higher prevalence in multihousehold dogs, significant differences were found for Ancylostomatidae and* T. vulpis* infections. It might be that in the presence of multiple pets, environmental contamination with infective stages of these taxa occurs; dogs become more susceptible to infections and environmental contamination itself is higher and better maintained. In cats, the presence of endoparasites was associated only with their age, housing, and with the area they lived in. These findings are consistent with other studies considering parasitism as of primarily concern for younger dogs or cats [4, 5, 7, 14]. According to the univariate analysis, the overall prevalence of intestinal parasites in household cats shows statistically significant differences with cats that lived outside/with access to a garden (*P* = 50% in household* versus P* = 65.52% in outdoor). Living outdoors or having access to a garden seems to be a risk factor for* T. leonina* infection in cats, as similarly described by Näreaho et al. [15]. It could be partially due to the source of infection for this parasite that, in addition to larvated eggs, is represented by paratenic hosts that harbour somatic third-stage larvae [44]. As a consequence of their predatory behavior, domestic felines could be more susceptible to infections due to paratenic hosts when they have outdoor access.

Dogs and cats presenting gastrointestinal signs showed a prevalence of intestinal parasites close to 45%, which urges to differential diagnosis and periodic coprological examination. Prophylaxis against* D. immitis* showed ineffective in protecting dogs against gastrointestinal nematodes. For them, registered dosage of macrocyclic lactones used against heartworms must be too low and seasonal administration of the treatment to all sampled adults dogs proved insufficient to cover their exposition to other risk factors all over the year. The answers to our questionnaire, specifically designed to understand owner's awareness and information about canine and feline gastrointestinal parasites, showed that they knew but few aspects of the parasite biology. In fact, more than 71.89% of them indicated that fecal contamination can cause gastrointestinal parasite infection, and thus they were probably aware of the importance of reducing environmental fecal voiding. Nonetheless, 39.10% of them gave a negative answer or no answer at all. As to possible lasting environmental contamination due to infected pet fecalization, 56.22% of total owners were not aware of it, and most of them probably did not consider preventing contact with intermediate/paratenic hosts as a possible prophylaxis against intestinal parasites infections. A higher number of owners (48.11%) correctly answered affirmatively when asked about the possibility of transmission of intestinal parasites to puppies/kittens by infective milk of bitches/queens. They may be more stressed by clinicians on the importance of intestinal parasite infections in puppies and kittens than in adult dogs and cats. Concerning their awareness of risks for human health from canine and feline intestinal parasites, 50.81% declared that intestinal parasites of dogs and cats do not represent any kind of risk for human health or that they did not know about the issue. Further, 26.37% out of 90 owners informed about human health risks stated that they could not name possible diseases, thus confirming that they did not know what proper behavior is necessary to reduce zoonotic risks.

Overall, these results indicated that owners needed more and clear information about zoonotic potential of intestinal parasites, and that the veterinarians can be of extreme importance in this process.

## 5. Conclusion

Results of this survey showed that intestinal parasites are still a common finding in owned dogs and cats not to be underestimated in both metropolitan and micropolitan areas, even if the latter indicated higher pet infection prevalence. Further, when a dog or a cat is presented to clinical examination on account of gastrointestinal signs, intestinal parasite infection should be considered as a possible differential diagnosis. This condition can be asymptomatic and can even affect animals under proper prophylaxis against* D. immitis*; thus, even apparently fit and healthy pets should be submitted to annual or biannual fecal examination. Clinicians should also consider that younger patients that live in micropolitan areas are the most susceptible to parasite infections. The zoonotic parasites* T. canis* and* T. cati*,* T. vulpis*, Ancylostomatidae, and* G. duodenalis* assemblage A resulted to be the most common species in owned pets. In any case, veterinaries clearly play a key role in increasing awareness and knowledge of pet owners about canine and feline gastrointestinal parasites as to their infection routes, proper monitoring, and correct behavior to avoid potential zoonotic risks.

## Figures and Tables

**Table 1:** Prevalence (%) and confidence interval (CI) of intestinal parasites in 253 owned dogs and 156 owned cats in northern Italy.

Parasites	Metropolitan area	Micropolitan area 1	Micropolitan area 2
	% (CI)	% (CI)	% (CI)
Dogs			
* Toxocara canis *	4.48	22.22	9.30
	(2.09–10.86)	(10.77–36.68)	(3.04–15.57)
* Toxascaris leonina *	0	3.70	0
	(0-0)	(0–8.91)	(0-0)
Ancylostomatidae	0.97	5.56	3.49
	(0.17–5.29)	(0–11.87)	(0–7.45)
* Trichuris vulpis *	6.08	11.11	5.81
	(3.33–13.37)	(2.45–19.77)	(0.77–10.86)
* Strongyloides stercoralis *	1.94	0	0
	(0.53–6.8)	(0-0)	(0-0)
* Eucoleus aerophilus *	0.97	0	0
	(0.17–5.29)	(0-0)	(0-0)
* Dipylidium caninum *	0	2.86	0
	(0)	(0.10–5.62)	(0-0)
* Cystoisospora *sp.	0.97	3.70	6.98
	(0.17–5.29)	(0–8.91)	(1.48–12.47)
* Giardia duodenalis *	16.05	20.37	25.58
	(10.56–24.85)	(9.27–31.47)	(16.17–34.99)
Overall prevalence	28.16	57.41	43.02
	(20.38–37.51)	(43.78–71.03)	(32.35–53.70)
Cats			
* Toxocara cati *	5.62	5.26	22.39
	(2.42–12.49)	(0–16.32)	(12.41–32.37)
* Toxascaris leonina *	0	5.26	8.96
	(0-0)	(0–16.32)	(2.12–15.80)
* Ancylostomatidae *	1.12	0	2.08
	(0.02–6.09)	(0-0)	(0–6.27)
* Trichuris vulpis *	0	0	2.08
	(0-0)	(0-0)	(0–6.27)
* Dipylidium caninum *	0	2.86	4.48
	(0-0)	(0.10–5.62)	(0–9.43)
* Spirometra *	1.12	0	0
	(0.02–6.09)	(0-0)	(0-0)
* Cystoisospora *sp.	1.12	5.26	4.17
	(0.02–6.09)	(0–16.32)	(0–10.03)
* Toxoplasma-*like	1.12	0	0
	(0.02–6.09)	(0-0)	(0-0)
* Giardia duodenalis *	22.47	36.84	25.00
	(15.04–32.18)	(12.96–60.73)	(12.29–37.71)
Overall prevalence	32.58	47.37	60.42
	(23.74–42.86)	(22.64–72.09)	(46.07–74.77)

**Table 2:** Univariate analysis of risk factors for intestinal parasites in dogs and cats presenting at two veterinary practices in northern Italy.

Variable	Risk factor	OR	95% CI	*P* value
Dogs				
Habitation	Metropolitan	1.00		0.006
	Micropolitan area 1	3.476	1.632–7.403	0.001
	Micropolitan area 2	1.947	0.986–3.845	0.055
Age	≤12 months	1.00		NA
	>12 months	0.362	0.205–0.639	0.000
Gender	Male	1.00		NA
	Females	0.756	0.433–1.320	0.325
Size	Small	1		0.917
	Medium	0.937	0.451–1.946	0.861
	Large	1.165	0.439–3.092	0.760
Housing	Household	1		NA
	Outdoor	0.827	0.364–1.880	0.651
Husbandry	Single-dog-household	1		NA
	Multiple-dog-household	2.059	1.047–4.051	0.036
Cats				
Habitation	Metropolitan	1.00		0.008
	Micropolitan area 1	2.100	0.730–6.039	0.169
	Micropolitan area 2	3.561	1.601–7.924	0.002
Age	≤12 months	1.00		NA
	>12 months	0.347	0.168–0.716	0.004
Gender	Males	1.00		NA
	Female	0.809	0.401–1.631	0.553
Housing	Outdoor/household	1		NA
	Household	0.526	0.195–1.424	0.006
Husbandry	Single-cat-household	1		NA
	Multiple-cat-household	1.153	0.437–3.039	0.774

OR: odds ratio.

(CI) 95%: confidence interval.

**Table 3:** Final multivariate analysis of risks factors associated with intestinal parasites in dogs and cats presenting at two veterinary practices in northern Italy.

Variable	Risk factor	OR	95% CI	*P* value
Dogs				
Age	>12 months	1.00		NA
	≤12 months	0.445	0.222–0.894	0.023
Husbandry	Single-dog-household	1		NA
	Multiple-dog-household	2.240	1.115–4.498	0.023
Cats				
Habitation	Metropolitan area	1.00		0.011
	Micropolitan area 1	2.279	0.762–6.814	0.141
	Micropolitan area 2	3.510	1.536–8.020	0.003
Age	>12 months	1.00		NA
	≤12 months	0.348	0.163–0.742	0.006

OR: odds ratio.

(CI) 95%: confidence interval.

**Table 4:** Prevalence (%) and 95% CI (min–max) of intestinal parasites in dogs and cats by individual features and management.

Parasites	Gender		Age		Housing		Husbandry	
	Male	Female	≤12 months	>12 months	Household	Outdoor	Single	Multiple
Dogs	(*n* = 116)	(*n* = 92)	(*n* = 92)	(*n* = 116)	(*n* = 56)	(*n* = 84)	(*n* = 74)	(*n* = 66)
* Toxocara canis *	13.79	7.61	20.65**	3.45**	10.71	16.67	10.81	18.18
	(7.52–20.07)	(2.19–13.03)	(12.38–28.92)	(0.13–6.77)	(2.61–18.81)	(8.70–24.64)	(3.74–17.88)	(8.88–27.48)
* Toxascaris leonina *	0.86	1.09	1.09	0.86	0	2.38	1.35	1.52
	(0–2.54)	(0–3.21)	(0–3.21)	(0–2.54)	—	(0–5.64)	(0–3.98)	(0–4.47)
Ancylostomatidae	3.45	3.26	3.26	3.45	1.79	5.95	0**	9.09**
	(0.13–6.77)	(0–6.89)	(0–6.89)	(0.13–6.77)	(0–5.26)	(0.89–11.01)	—	(2.15–16.03)
* Trichuris vulpis *	5.17	8.70	3.26	9.48	5.36	9.52	2.70*	13.64*
	(1.14–9.20)	(2.94–14.45)	(0–6.89)	(4.15–14.81)	(0–11.26)	(3.24–15.80)	(0–6.39)	(5.36–21.92)
* Eucoleus aerophilus *	0	1.09	0	0.86	0	0	0	0
	—	(0–3.21)	—	(0–2.54)	—	—	—	—
* Dipylidium caninum *	1.72	2.17	2.17	1.72	5.36	1.19	2.70	3.03
	(0–4.09)	(0–5.15)	(0–5.15)	(0–4.09)	(0–11.26)	(0–3.51)	(0–6.39)	(0–7.17)
* Cystoisospora *sp.	3.45	5.43	8.70**	0.86**	10.71*	2.38*	5.41	6.06
	(0.13–6.77)	(0.80–10.07)	(2.94–14.45)	(0–2.54)	(2.61–18.81)	(0–5.64)	(0.26–10.56)	(0.30–11.82)
* Giardia duodenalis *	21.55	18.48	27.17*	14.66*	26.79	21.43	22.97	24.24
	(14.07–29.03)	(10.55–26.41)	(18.08–36.26)	(8.22–21.09)	(15.19–38.39)	(12.65–30.21)	(13.39–32.55)	(13.90–34.58)
Overall prevalence	44.83	38.04	55.43**	29.31**	50	44.83	40.54*	57.58*
	(35.78–53.88)	(28.12–47.96)	(45.28–65.59)	(21.03–37.59)	(36.90–63.15)	(25.58–64.08)	(29.35–51.73)	(45.66–69.50)
Cats	(*n* = 62)	(*n* = 65)	(*n* = 61)	(*n* = 66)	(*n* = 38)	(*n* = 29)	(*n* = 31)	(*n* = 36)
* Toxocara cati *	12.90	12.31	19.67*	6.06*	23.68	20.69	19.35	25.00
	(4.56–21.25)	(4.32–20.29)	(9.70–29.65)	(0.30–11.82)	(10.16–37.20)	(5.95–35.43)	(5.44–33.26)	(10.85–39.15)
* Toxascaris leonina *	3.23	6.15	9.84**	0**	2.63*	17.24*	9.68	8.33
	(0–7.62)	(0.31–12.00)	(2.36–17.31)	—	(0–7.72)	(3.49–30.99)	(0–20.09)	(0–17.36)
Ancylostomatidae	1.61	0	1.64	0	2.63	0	0	2.78
	(0–4.75)	—	(0–4.83)	—	(0–7.72)	—	—	(0–8.15)
* Trichuris vulpis *	1.61	0	0	1.52	0	3.45	0	2.78
	(0–4.75)	—	—	(0–4.46)	—	(0–10.09)	—	(0–8.15)
* Dipylidium caninum *	3.23	1.54	0	4.55	7.89	0	3.23	5.56
	(0–7.62)	(0–4.53)		(0–9.57)	(0–16.46)	—	(0–9.45)	(0–13.05)
* Spirometra *sp.	1.61	0	1.64	0	0	0	0	0
	(0–4.75)	—	(0–4.83)	—	—	—	—	—
* Cystoisospora *sp.	1.61	4.62	4.92	1.52	2.63	6.90	0	8.33
	(0–4.75)	(0–9.72)	(0–10.34)	(0–4.46)	(0–7.72)	(0–16.12)	—	(0–17.36)
* Toxoplasma*-like	0	1.54	0	1.52	0	0	0	0
	—	(0–4.53)	—	(0–4.46)	—	—	—	—
* Giardia duodenalis *	29.03	26.15	32.79	22.73	23.68	34.48	35.48	22.22
	(17.73–40.33)	(15.47–36.84)	(21.01–44.57)	(12.62–32.84)	(10.16–37.20)	(17.18–51.78)	(18.64–52.32)	(8.64–35.80)
Overall prevalence	46.77	41.54	57.38**	31.82**	50	65.52	54.84	58.33
	(34.35–59.19)	(29.56–53.52)	(44.97–69.79)	(20.58–43.06)	(34.10–65.90)	(48.22–82.82)	(37.32–72.36)	(42.22–74.44)

(CI) 95%: confidence interval of the prevalence.

**Chi-Square test. *P* value < 0.01.

*Chi-Square test. *P* value < 0.05.

**Table 5:** Frequency of pets (dogs or cats) infected or not infected by intestinal parasites in northern Italy according to gastrointestinal symptoms (presence or absence).

	Infection	No infection	*P* value*
Dogs			
Symptomatic	21	23	0.85
Asymptomatic	48	48	
Cat			
Symptomatic	10	13	0.128
Asymptomatic	28	16	

*Fisher's exact *P* value.

**Table 6:** Frequency of pets (dogs or cats) infected by intestinal parasites in northwestern Italy by features of their owners (number 207).

Owner features	Frequency of pets		*P* value*
	Positive *n* (%)	Negative *n* (%)	
Gender			
Female	75 (36.94)	66 (32.51)	0.24
Male	29 (14.28)	33 (16.25)	
Age			
≤40 years old	65 (32.02)	66 (32.51)	0.31
>40 years old	39 (19.21)	33 (16.25)	
Educational qualification			
Secondary school certificate	38 (18.71)	33 (16.25)	0.37
Intermediate school certificate/academic degree	66 (32.51)	66 (32.51)	
Family components			
≤2	42 (20.58)	47 (23.03)	0.20
>2	62 (30.39)	53 (25.98)	
Presence of young <15 years old			
Not	66 (32.35)	68 (33.33)	0.29
Yes	38 (18.62)	32 (15.68)	

*Fisher's exact *P* value.
